# Multi-omics profile of the mouse dentate gyrus after kainic acid-induced status epilepticus

**DOI:** 10.1038/sdata.2016.68

**Published:** 2016-08-16

**Authors:** Marijn Schouten, Pascal Bielefeld, Silvina A. Fratantoni, Chantal J. Hubens, Sander R. Piersma, Thang V. Pham, Rob A. Voskuyl, Paul J. Lucassen, Connie R. Jimenez, Carlos P. Fitzsimons

**Affiliations:** 1 Neuroscience Program, Swammerdam Institute for Life Sciences, Faculty of Sciences, University of Amsterdam, Science Park 904, Amsterdam 1098XH, The Netherlands; 2 Oncoproteomics Laboratory, Cancer Center, Free University Amsterdam, De Boelelaan 1117, Amsterdam 1081HV, The Netherlands; 3 Division of Pharmacology, LACDR, Leiden University, Einsteinweg 55, Leiden 2333 CC, The Netherlands; 4 Foundation of Epilepsy Institutes of The Netherlands (SEIN), Achterweg 5, Heemstede 2103 SW, The Netherlands

**Keywords:** Molecular neuroscience, Proteomic analysis, Microarray analysis, Reverse transcription polymerase chain reaction, Epilepsy

## Abstract

Temporal lobe epilepsy (TLE) can develop from alterations in hippocampal structure and circuit characteristics, and can be modeled in mice by administration of kainic acid (KA). Adult neurogenesis in the dentate gyrus (DG) contributes to hippocampal functions and has been reported to contribute to the development of TLE. Some of the phenotypical changes include neural stem and precursor cells (NPSC) apoptosis, shortly after their birth, before they produce hippocampal neurons. Here we explored these early phenotypical changes in the DG 3 days after a systemic injection of KA inducing status epilepticus (KA-SE), in mice. We performed a multi-omics experimental setup and analyzed DG tissue samples using proteomics, transcriptomics and microRNA profiling techniques, detecting the expression of 2327 proteins, 13401 mRNAs and 311 microRNAs. We here present a description of how these data were obtained and make them available for further analysis and validation. Our data may help to further identify and characterize molecular mechanisms involved in the alterations induced shortly after KA-SE in the mouse DG.

## Background & Summary

TLE is a serious neuropathological condition hallmarked by chronic spontaneous recurrent seizures, which can originate from altered hippocampal circuit characteristics. Strong initial seizures, such as those seen during SE, have previously been proposed as a driving force in the development of TLE^1^. The current view on TLE development, proposes a number of causal changes in hippocampal structure including DG granule cell layer dispersion^2^, mossy fiber sprouting^3^, and the induction of aberrant hippocampal neurogenesis^4,5^. These abnormalities can be modeled using convulsant treatments such as e.g., KA administration^4^. Concerning aberrant neurogenesis, multiple cellular phenotypes have been linked to the nature of newly born aberrant neurons including alterations in levels of NSPC proliferation, and subsequent changes in the levels of apoptosis, differentiation, integration and ectopic positioning of their progeny^4–6^.

Although the aforementioned structural alterations following KA administration have been widely documented, the molecular mechanisms involved are less well characterized. For instance, molecular events contributing to lower levels of NSPC apoptosis following KA-SE are poorly understood. Indeed, although others have previously characterized executioner caspases to be crucial mediators of SE-induced changes in apoptosis^7^, the upstream molecular events controlling caspase activation were unknown. Our specific aim was to understand the molecular mechanisms that may underlie altered apoptosis levels in the DG following KA-induced status epilepticus (KA-SE).

A number of previous studies have applied -omics approaches to describe changes in DG gene expression (shortly) after KA-SE at the level of mRNA^8^. These results should be interpreted carefully, since previous studies have indicated a poor correlation of mRNA expression to their corresponding protein levels^9,10^, suggesting a significant contribution of post-transcriptional mechanisms to gene regulation. Amongst the many factors which could possibly contribute to post-transcriptional gene expression regulation, microRNAs are a particular class of molecules whose expression could hinder target mRNA translation into protein^11^. Indeed, previous studies have also approached TLE from the perspective of microRNA expression^12^. There were, to our knowledge, no previous records of an experimental design allowing simultaneous protein, mRNA and microRNA expression measurements shortly after KA-SE in the DG. Accordingly, we set out in such a multi-omics approach to explore in particular alterations in mitochondrial function-related protein expression, which could be affected shortly after KA-SE in the DG. We used systemic injections of KA to ensure an equal distribution of the chemo-convulsant to both hemispheres and thus both dentate gyri. In addition, to specifically identify the molecular mechanisms of KA-SE on the DG, we conducted our measurements on DG enriched samples^13^. To this end, we used the left DG to analyze protein expression and the right DG for RNA expression analyses. The latter included both mRNA and microRNA measurements.

Our multi-omics approach allowed us to identify a novel role for cooperative microRNA-mediated regulatory action on the expression of the pro-apoptotic protein BCL2L13 and downstream molecular events, including caspase-3 activity^14^. The use of 3 animals per experimental group, although enough to reach sufficient statistical power for further analysis, maybe be considered a limitation of our study. The dataset described here may allow the identification and further characterization of molecular mechanisms that could help to understand the numerous phenotypical alterations taking place in the DG shortly after KA-SE.

## Methods

### Animals

**Figure 1:**
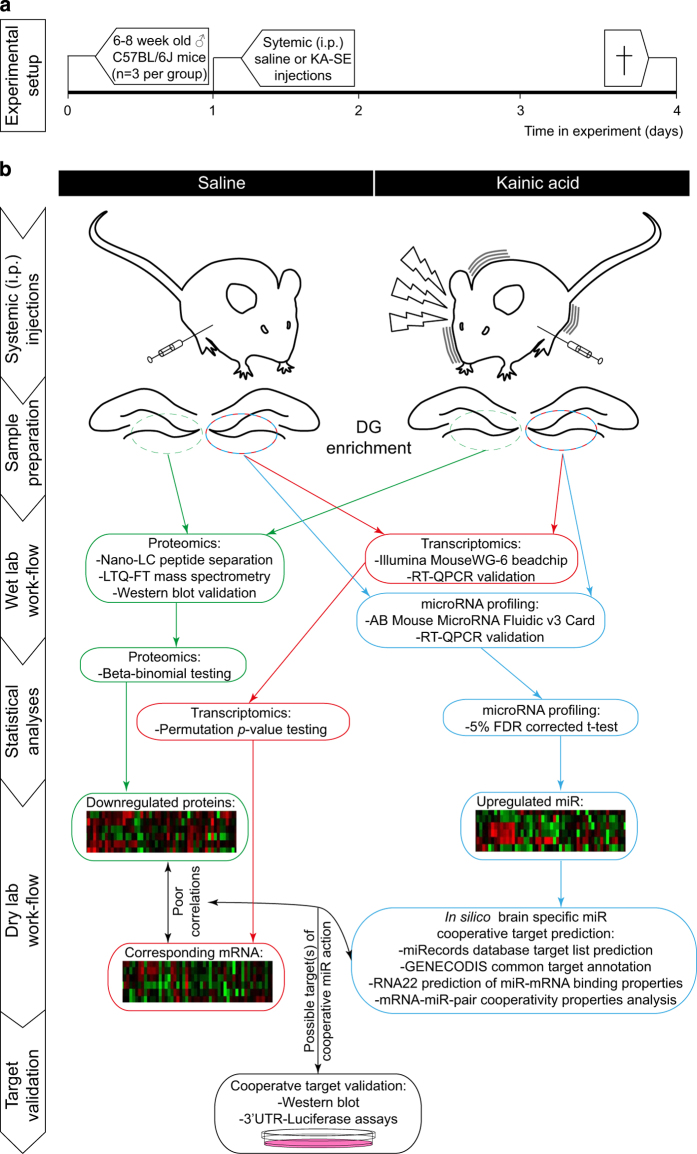
Schematic depiction of the experimental setup and subsequent workflow used to obtaining multi-omics data of DG tissue, 3 day after KA-SE. (**a**) Schematic illustration showing the timeline of experimental procedures on the animals. (**b**) Illustration on the experimental procedures, sample preparation, wet-lab workflow and statistical analysis steps carried out to produce proteomics, transcriptomics and microRNA profiles 3 days after KA-SE in the DG. The dry-lab workflow and target validation procedures are examples of how this data set was used by Schouten *et al.*^14^ to identify molecular mechanisms underlying alterations in NSPC apoptosis following KA-SE, yet not further discussed here.

SE-related aberrant neurogenesis properties can be induced in rodents by administering chemoconvulsants, such as Pilocarpine and KA^4,5,7,15,16^. In this particular study, SE was induced in C57BL/6j mice (*n*=3/group) by repeated (30 min interval) i.p. low-dose (initial dose 24 mg kg^−1^ and subsequent doses 10 mg kg^−1^) KA injections according to a previously described protocol by Hellier *et al.*^17^, modified for mice^18^. Likewise, after each KA injection, seizure classes were scored following a modified version of Racine’s scale^18^, and only animals scored for >5 min long class IV-V seizures were included for further analysis. Matching quantities of saline were injected in control animals. This systemic KA exposure approach allows for a low KA distribution variability between hemispheres^19^. Accordingly, this approach was chosen to allow for comparable KA levels to either hemisphere. Indeed, the experimental workflow depicted in Fig. 1 and Table 1, shows how the left hippocampus of each animal was used for the analysis of protein expression and the right for RNA expression, allowing good correlation analyses. Depending on the particular phenotype studied, one can vary the time of animal sacrifice after KA-SE. Since previous reports have identified most newly born neurons to undergo selection through apoptosis within the first week after their birth^7,20^, we opted for a shorter time point of 3 days after KA-SE to assess possible alterations in apoptosis-related proteins (Fig. 1).

### Sample preparation

To further enrich our samples with proteins and RNA originating from hippocampal neurogenic cells, we micro-dissected tissue from the DG. We excluded contribution from other brain areas to our sample content by micro-dissecting the DG using the hippocampal fissure as reference, as described before^13^. Briefly, before the procedure the animals were deeply anesthetized by IP injection of sodium pentobarbital (Euthasol 20%, ASTfarma BV, Oudewater, The Netherlands) and decapitated. The brains were rapidly removed from the skull and placed in a ice-cold solution A containing (in mM): NaCl, 124; KCl, 2.5; NaH2PO4, 1.25; CaCl2, 1; MgCl2, 1; NaHCO3, 25; D-Glucose, 10, that was constantly bubbled with 95%O2/5% CO2. Brains were then sliced coronally in 300 μm-thick slices using a Leica VT1000S vibratome. Coronal slices containing the dentate gyrus were collected on ice-cold solution A and the dentate gyrus was dissected from the hippocampus and the surrounding ventricular tissue under a dissecting microscope, following the hippocampal fissure separating the dentate gyrus from the rest of the hippocampus. A cut was then place between the dentate gyrus and the ventricular surface on one side and the CA3 region on the other. The dissected dentate gyrus was kept in ice-cold solution A until further processing.

For proteomic analysis, the protein of the left DG-enriched tissue samples were first captured in lysis buffer (per 100 mg tissue, one ml buffer containing 7 M urea, 2 M thiourea, 4% (w/v) CHAPS, and 10 μl ml^−1^ protease inhibitor mix (Amersham Biosciences, Piscataway, NJ, USA), and protein concentration measurements were carried before storage at −80 °C until further use. Matched quantities (30 μg) of protein sample were loaded on NuPAGE 4–12% gradient Bis-Tris gels (Invitrogen). Proteins were then digested in the gel using trypsin, which was subsequently sliced into 10 bands per lane prior to protein purification, as previously described^21^. Gels were washed and dehydrated once in 50 mM ammonium bicarbonate (ABC) and twice in 50 mM ABC/50% acetonitrile (ACN). Cysteine bonds were reduced by incubation with 10 mM DTT/50 mM ABC at 56 °C for 1 h and alkylated with 50 mM iodoacetamide/50 mM ABC at room temperature (RT) in the dark for 45 min. After washing sequentially with ABC and ABC/50% ACN, the whole gel was sliced in 10 bands of equal width for each lane. Gel bands were sliced up into approximately 1-mm^3^ cubes and collected in tubes, washed in ABC/ACN and dried in a vacuum centrifuge. Gel cubes were incubated overnight at 23 °C with 6.25 ng ml^−1^ trypsin and covered with ABC to allow digestion. Peptides were extracted once in 1% formic acid and twice in 5% formic acid/50% ACN. The volume of the peptide extract was reduced to 60 μl in a vacuum centrifuge and filtered using a 0.45 μm filter to remove gel particles and contaminants prior to LC-MS analysis. Subsequently, peptides were separated by an Ultimate 3000 nanoLC system (Dionex LC-Packings, Amsterdam, The Netherlands) equipped with a 20 cm×75 μm ID fused silica column custom packed with 3 μm 120 Å ReproSil Pur C18 aqua (Dr Maisch GMBH, Ammerbuch-Entringen, Germany). After injection, peptides were trapped at 6 μl/minute in 1.6% acetonitrile+0.05% formic acid on a 1 cm×100 μm ID precolumn packed with 5 μm ReproSil Pur C18 aqua. Peptides were separated in a 60 min gradient (8–32% acetonitrile in 0.05% formic acid) at 300 nl/min. followed by washing (72% acetonitrile in 0.05% formic acid) and equilibration (4% acetonitrile in 0.05% formic acid). The inject-to-inject time was 90 min. Purified fractions of protein per experimental replicate were now ready to undergo mass spectrometry analysis.

For transcriptomics and microRNA profiling, total RNA was isolated from the right DG enriched tissue samples using a TRIzol based RNA extraction protocol according to the manufacturers description. Purified RNA samples were analyzed for RNA concentrations using a Nanodrop and analyzed for RNA Integrity Numbers using a Nano Lab-on-Chip and Agilent Bioanalyzer platform. All total RNA sample RNA Integrity Numbers (RIN) were >8. Total RNA samples were then stored at −80 °C until further usage.

### Mass spectrometry for proteomics

Intact peptide mass spectra and fragmentation spectra were acquired on a LTQ-FT hybrid mass spectrometer (Thermo Fisher, Bremen, Germany). Intact masses were measured at a resolution of 50 000 in the ICR cell using a target value of 1×106 charges. In parallel, following an FT prescan, the top 5 peptide signals (charge-states 2+ and higher) were submitted to MS/MS in the linear ion trap (3 amu isolation width, 30 ms activation, 35% normalized activation energy, Q-value of 0.25 and a threshold of 5000 counts). Dynamic exclusion was applied with a repeat count of 1 and an exclusion time of 30 s.

### Protein identification and quantification

MS/MS spectra were searched against IPI mouse database 3.59 (56692 entries) using Sequest (version 27, rev 12) with a maximum allowed deviation of 10 ppm for the precursor mass and 1 amu for fragment masses. Methionine oxidation and cysteine carboxamidomethylation were allowed as variable modifications, two missed cleavages were allowed. Scaffold 2.06.01 (Proteome software, Portland, OR) was used to organize the gel-slice data and to validate peptide and protein identifications. Identifications with a Peptide Prophet probability> 95% were retained. Subsequently, protein identifications with a ProteinProphet probability of >99% with 2 peptides or more in at least one of the samples were retained. Proteins that contained similar peptides and could not be differentiated based on MS/MS analysis alone were grouped. For quantitative protein analysis across samples, spectral counts (number of identified MS/MS spectra for each protein) were normalized on the sum of the spectral counts per biological sample. Differential analysis of samples was performed using the BetaBinominal test as described previously^22,23^.

### Illumina mouseWG-6 beadchip hybridizations for transcriptomics

500 ng of each total RNA sample was processed into biotinylated cRNA using an Illumina TotalPrep RNA amplification Kit (Ambion, Life Technologies). Subsequently, 1500 ng of biotinylated cRNA of each experimental replicate was hybridized onto MouseWG-6 beadchips (Illumina) according to the manufacturers’ protocol.

### Mouse microRNA fluidic v3.0 cards for microRNA profiling

800 ng of each total RNA sample was processed into cDNA using Taqman microRNA RT Kit and Megaplex RT primers (Applied Biosystems), according to the manufacturers’ protocol. Subsequently, cDNA samples were combined with a Taqman Universal Master Mix prior to loading onto Mouse microRNA fluidic v3.0 cards (Applied Biosystems).

### Data analyses

Spectral counts were normalized against the sum spectral counts per biological sample. For the comparisons of data consisting of sample groups (*n* >1), various models are available to statistically test for significant differences in spectral count data, such as those obtained from mass spectrometry. Indeed, *G*-tests, *t*-tests, Fisher’s exact tests and the local-pooled-error technique have previously been used to identify differences in spectral counts of corresponding protein levels^23,24^. These models, however, fail to take into account variations within and in between sample groups. Accordingly, we opted to use a beta-binomial model to test for differences in protein abundances, taking these variations into account and thus resulting in an overall higher true detection rate, lower false positive rate and thus better experimental resolution^23^. In addition, this method allows for the comparison of protein levels when spectral counts are detected in at least one sample group (e.g., saline treatment replicates), but not the other (e.g., KA-SE treatment replicates)^23^. This software, used to test amongst others spectral counts of experimental setups with multiple experimental groups composed of multiple replicates, is freely available as an R software package from www.oncoproteomics.nl, and has been extensively described in Pham *et al.*^23^.

Concerning data analysis of transcriptomics, probe intensities from the Illumina mouseWG-6 beadchip arrays were analyzed in GenomeStudio. Firstly, transcript expression detection limits were analyzed for all samples. Detection *P*-values were calculated based on mismatch probe intensity, negative control probe intensity and stringency. Detection *P*-value cut-off was set to <0.05 as a quality control and inclusion criterion for further statistical testing. Subsequently, data were background-corrected, transformed and normalized as described before^25^. Permutation *P*-value calculation was used to determine significant differences between saline and KA-SE treatments (*P*<0.01).

The detection limit of each microRNA was determined by including microRNA with a raw Ct value <35 for further statistical analysis. Data were subsequently normalized against RNU6B expression levels allowing a ΔΔCt-based conversion to fold change expression levels of each microRNA between treatment groups. A 5% false discovery rate corrected *t*-test was used to determine statistical differences (*P*<0.05 and fold change≥1.5).

## Data Records

### Data record 1

The mass spectrometry proteomics data have been deposited in the ProteomeXchange Consortium via the PRIDE partner repository^26^ with the dataset identifier PXD003744 (Data citation 1).

### Data record 2

The microarray transcriptomics data have been deposited in NCBI’s GEO repository with the dataset identifier GSE79129 (Data citation 2).

### Data record 3

The RT-QPCR microRNA profiling data have been deposited in NCBI’s GEO repository with the dataset identifier GSE79131 (Data citation 3).

## Technical Validation

### Proteomic data

**Figure 2:**
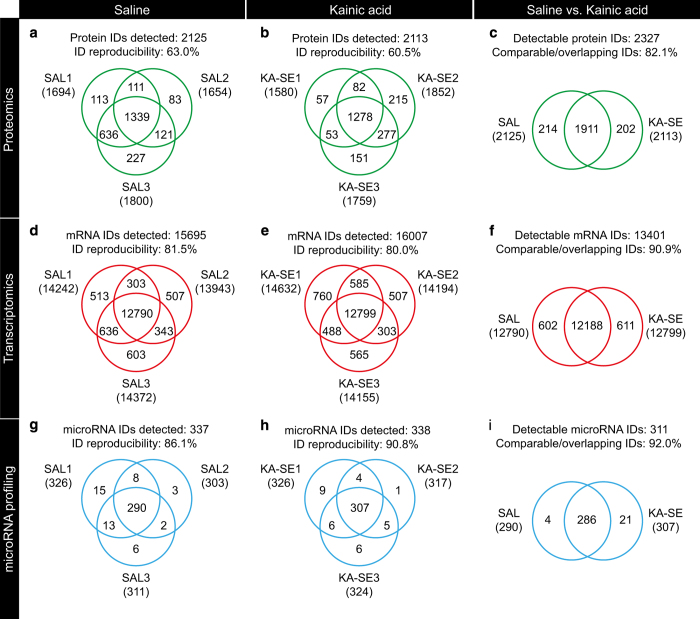
Experimental replicates and reproducibility in multi-omics profiling. Venn diagrams of all experimental replicates and groups showing within group reproducibility of proteomics (**a**,**b**), transcriptomics (**d**,**e**) and microRNA profiling (**g**,**h**) identities (IDs) and subsequent in between group comparability/overlap of IDs for proteomics (**c**), transcriptomics (**f**) and microRNA profile (**i**) data.

Within the saline sample group, of the total 2125 proteins identified, 1339 could be identified in all three samples resulting in an ID reproducibility of 63.0%, as depicted in the Venn diagram of Fig. 2a. Likewise, within the KA-SE sample group, of the total 2113 proteins identified, 1278 could be identified in all three samples resulting in an ID reproducibility of 60.5%, as depicted in the Venn diagram of Fig. 2b. These ID reproducibility values were accompanied by calculated coefficients of variance of 21.5 and 24.6% for saline and KA-SE, respectively. These values meet the required standards for proteomic analysis suggested in the literature^27,28^. Accordingly, the Venn diagram comparison between experimental groups shows 82.1% overlapping/comparable IDs as depicted in Fig. 2c. To further validate the mass spectrometry based observations western blot analysis was performed. Immunolabelling was performed on the same samples to validate the expression levels of proteins of interest (BAX and BCL2L13), normalized to beta-actin protein expression levels. As previously described in Schouten *et al.*^14^, we found the western blot data to support the proteomics data for the expression levels of BAX and BCL2L13 proteins.

### Transcriptomic data

Venn diagrams of saline group replicates (Fig. 2d) and KA-SE group replicates (Fig. 2e) show good (>80%) within group reproducibility percentages. Furthermore, a Venn diagram of the experimental groups with overlapping/comparable IDs is shown in Fig. 2f, depicting a good between-group comparison percentage (>90%). RT-QPCR was performed on the same samples to validate expression levels of transcripts of interest (BAX and BCL2L13), normalized to alpha-tubulin mRNA expression levels. As previously described in Schouten *et al.*^14^, we found the RT-QPCR data to support the transcriptomics data for the expression levels of BAX and BCL2L13 mRNA.

### MicroRNA profiling data

Venn diagrams of saline group replicates (Fig. 2g) and KA-SE group replicates (Fig. 2h) show good (>85%) within group reproducibility percentages. Furthermore, a Venn diagram of the experimental groups with overlapping/comparable IDs is shown in Fig. 2i, depicting a good between-group comparison percentage (>90%). RT-QPCR was performed on the same samples to validate expression levels of microRNA of interest (microRNA-124 and microRNA-137), normalized to RNU6B expression levels. As previously described in Schouten *et al.*^14^, we found the RT-QPCR data to support the microRNA profiling data for the expression levels of microRNA-124 and microRNA-137.

## Usage Notes

The beta-binomial software is freely available as an R software package from www.oncoproteomics.nl, and extensively described by Pham *et al.*^23^.

## Additional Information

**How to cite this article:** Schouten, M. *et al.* Multi-omics profile of the mouse dentate gyrus after kainic acid-induced status epilepticus. *Sci. Data* 3:160068 doi: 10.1038/sdata.2016.68 (2016).

## Supplementary Material



## Figures and Tables

**Table 1:** Information on samples and their related datasets stored in online repositories.

**Subjects**	**Drug treatment**	**Tissue**	**Sample treatment**	**Data collection**	**Data**
Mouse1–3	Saline treatment	Left DG dissection	Protein extraction	Mass spectrometry	PXD003744
Mouse1–3	Saline treatment	Right DG dissection	Total RNA extraction	Illumina mouseWG-6 beadchip hybridization	GSE79129
Mouse1–3	Saline treatment	Right DG dissection	Total RNA extraction	Mouse microRNA fluidic v3.0 card	GSE79131
Mouse4–6	KA-SE treatment	Left DG dissection	Protein extraction	Mass spectrometry	PXD003744
Mouse4–6	KA-SE treatment	Right DG dissection	Total RNA extraction	Illumina mouseWG-6 beadchip hybridization	GSE79129
Mouse4–6	KA-SE treatment	Right DG dissection	Total RNA extraction	Mouse microRNA fluidic v3.0 card	GSE79131
